# NEK2 plays an active role in Tumorigenesis and Tumor Microenvironment in Non-Small Cell Lung Cancer: Retraction

**DOI:** 10.7150/ijbs.75303

**Published:** 2022-06-07

**Authors:** Rui Bai, Cheng Yuan, Wenjie Sun, Jianguo Zhang, Yuan Luo, Yanping Gao, Yangyi Li, Yan Gong, Conghua Xie

**Affiliations:** 1 Department of Radiation and Medical Oncology, Zhongnan Hospital of Wuhan, University, Wuhan, Hubei 430071, China.; 2 Department of Biological Repositories, Zhongnan Hospital of Wuhan, University, Wuhan, Hubei 430071, China.; 3 Tumor Precision Diagnosis and Treatment Technology and Translational Medicine Hubei Engineering Research Center, Zhongnan Hospital of Wuhan University, Wuhan, Hubei 430071, China.; 4 Hubei Key Laboratory of Tumour Biological Behaviors, Zhongnan Hospital of Wuhan University, Wuhan, Hubei 430071, China.; 5 Hubei Cancer Clinical Study Center, Zhongnan Hospital of Wuhan University, Wuhan, Hubei 430071, China.

The authors recently found that some results were not repeatable in the cell lines and needed further verifications. After careful consideration, the authors decided to retract this article. All authors have agreed to the retraction of the article, and apologize for any inconvenience caused.

